# Supplementary material for: Novel cancer subtyping method based on patient-specific gene regulatory network
Source: Sci Rep. 2021 Dec 8;11:23653. doi: 10.1038/s41598-021-02394-w (PMC8654869; doi:10.1038/s41598-021-02394-w)
Supplement: Supplementary file 1 — Supplementary Information 1. [file 41598_2021_2394_MOESM1_ESM.pdf]

Novel cancer subtyping method based on patient-specific molecular networks

Authors: Mai Adachi Nakazawa, Yoshinori Tamada, Yoshihisa Tanaka, Marie Ikeguchi, Kako Higashihara, Yasushi Okuno

All supplementary data is available at https://ytlab.jp/suppl/nakazawa2021/index.html

Supplement Information

S1 Methods

S1.1 Computational and statistical environment

The network estimations of STAD and BRCA, and ECv calculations were performed using the SHIROKANE supercomputer system at Human Genome Center, the Institute of Medical Science, the University of Tokyo. The network estimations of BRCA were performed using Fugaku supercomputer system at RIKEN in Kobe.

All statistical analyses were performed using Python unless stated otherwise. Hierarchical clustering was performed using the "ward" methods and Euclidean distance in Python library. The survival analysis was evaluated using the log-rank test in R package survival and Python library lifelines. Molecular function analysis was performed using Ingenuity Pathway Analysis (IPA)[1]. Network visualization and analysis were performed in Cytoscape[2].

S2 Result

S2.1 Dataset

These RNA-seq data were downloaded from UCSC Xena[3]. Clinical data were downloaded from GDC Data Portal at TCGA to evaluate the results of the analysis. Patients were selected for whom both the RNA-seq data of the tumor specimens and the clinical data (five years) for each cancer type were available. Next, the genes with a mean percentile under 15 were removed from the RNA-seq data of each dataset. Ultimately, 365 patients were selected for STAD, 692 patients for LUNG, and 1095 patients for BRCA. The preprocessed RNA-seq datasets comprised different sets of 17,450 genes.

S2.2 Network estimation

The NNSR algorithm determines the final network structure by extracting the edges whose estimated frequencies are greater than a given threshold. A threshold of 0.1 was used in our analysis, as in Tanaka et al. (2020)[4]. This algorithm repeatedly estimates subnetworks, including a thousand nodes, such that we first checked whether the algorithm produced stable networks. The network estimation was

conducted independently three times, and the concordance of the estimated edges was calculated between the two estimated networks as an indicator of robustness in the estimated networks, as in Tanaka et al. (2020)[4]. Consequently, when the number of iterations (T) of the subnetwork estimation was 100,000, as recommended by Tamada et al. (2011)[5], the concordance was less than 95%. However, since this is slightly below the necessary level of network stability, we used $T = 300{,}000$ and obtained a concordance of 96.3%, 95.6%, and 95.6%, for STAD, LUNG, and BRCA respectively (Table S1). These results suggested that the structures of the estimated networks were stable, and that T=300,000 was sufficient for our analysis. In STAD and LUNG datasets, the running time required for these network estimations were 22 hours 27 minutes 17 seconds at T = 300,000 using 64 CPU cores with SHIROKANE, and 11 hours 56 minutes 6 seconds, independently. In BRCA datasets, the running time required for it was 1 1hour 11 minutes 2 seconds at T = 300,000 using 6144 CPU cores with Fugaku.

S2.3 Comparison of the $\tilde{\Delta} ECv$ and FC distribution

The distributions between $\tilde{\Delta} ECv$ and $\log_2$ fold change (FC) were compared to present that only the limited edges show significant differences. The distribution of $\tilde{\Delta} ECv$ is much steeper than that of $\log_2$ FC. The FC for RNA-seq data is defined as one subtype/the rest of two subtypes. We overlapped $\tilde{\Delta} ECv$ and FC in the same histogram (Fig. 2c-e, Fig. S2).

S2.4 Hierarchical clustering for RNA-seq data

For LUNG and BRCA dataset, the top 310 and 347 genes showing the highest variances of the RNA-seq data were selected for hierarchical clustering, respectively, as the 250 edges with the ECv matrix in LUNG and BRCA were composed of 310 and 347 genes, respectively.

Table S1. Concordance of the estimated networks.

	$T = 100{,}000$	$T = 300{,}000$
STAD	92.7%	96.3%
LUNG	92.7%	95.6%
BRCA	N/A	95.6%

Table S2. The summarization of the number of patients across subtypes identified by the clustering of ECv matrix and RNA-seq data in the STAD dataset.

		RNA-seq		
		subtype 1A	subtype 2A	subtype 3A
ECv	subtype 1	91	8	15
	subtype 2	43	0	33
	subtype 3	77	24	74

Table S3. The summarization of the number of patients across the identified subtypes and their sex.

	male	female
subtype 1	79	34
subtype 2	0	76
subtype 3	161	14

Table S4. The summarization of the number of patients across the identified subtypes and their age at diagnosis

	Age at diagnosis
subtype 1	63.9
subtype 2	66.7
subtype 3	66.2

Table S5. The summarization of the number of patients across the identified subtypes and their tumor stage.

	stage i	stage ii	stage iii	stage iv
subtype 1	6	40	42	7
subtype 2	11	30	16	6
subtype 3	31	45	69	15

Table S6. The summarization of the number of patients across subtypes identified by the clustering of ECv matrix and RNA-seq data in the LUNG dataset.

			RNA-seq			
			subtype 1A	subtype 2A	subtype 3A	all
ECv	subtype 1	LUAD	4	0	0	4
		LUSC	224	0	0	224
	subtype 2	LUAD	0	51	57	108
		LUSC	0	10	3	13
	subtype 3	LUAD	1	48	266	315
		LUSC	3	7	18	28
	all		LUAD 5<br>LUSC 227	LUAD 99<br>LUSC 17	LUAD 323<br>LUSC 21	LUAD 427<br>LUSC 265

Table S7. The summarization of the number of patients across subtypes identified by the clustering of ECv matrix and RNA-seq data in the BRCA dataset.

		RNA-seq		
		subtype 1A	subtype 2A	subtype 3A
ECv	subtype 1	185	4	2
	subtype 2	0	333	91
	subtype 3	2	131	346

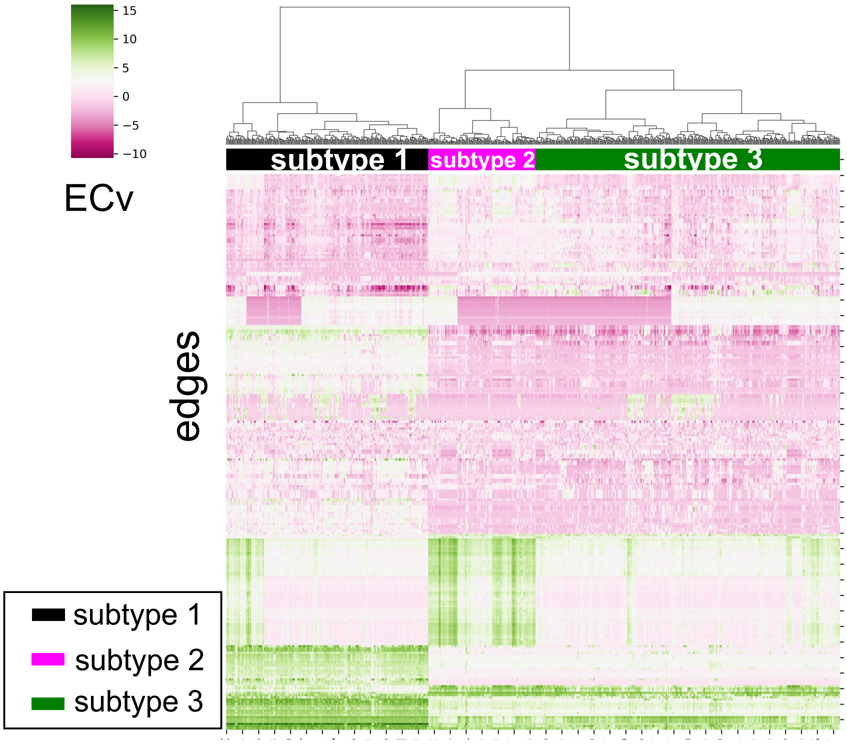

Figure S1. Heatmap showing hierarchical clustering for the ECv matrix in the LUNG dataset.

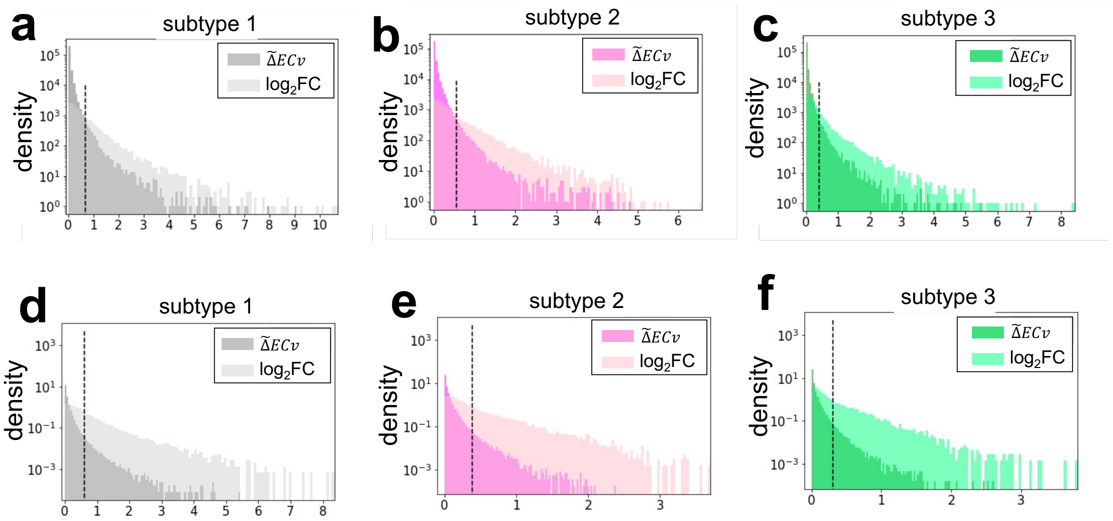

Figure S2. The distribution of $\widetilde{\Delta}ECv$ of edges and $\log_2$ fold change (FC) in genes in the LUNG datasets (a-c) and in the BRCA datasets (d-f) . Dashed line represents the $\widetilde{\Delta}ECv$ of the top 1.0% of total edges in every subtype.

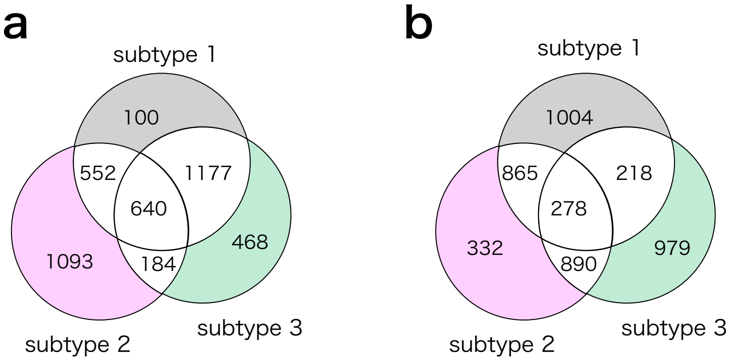

Figure S3. The Venn diagram represents the number of edges in the LUNG dataset (a) and in the BRCA dataset (b). Colored areas in the Venn diagram represent subtype-specific edges in each subtype.

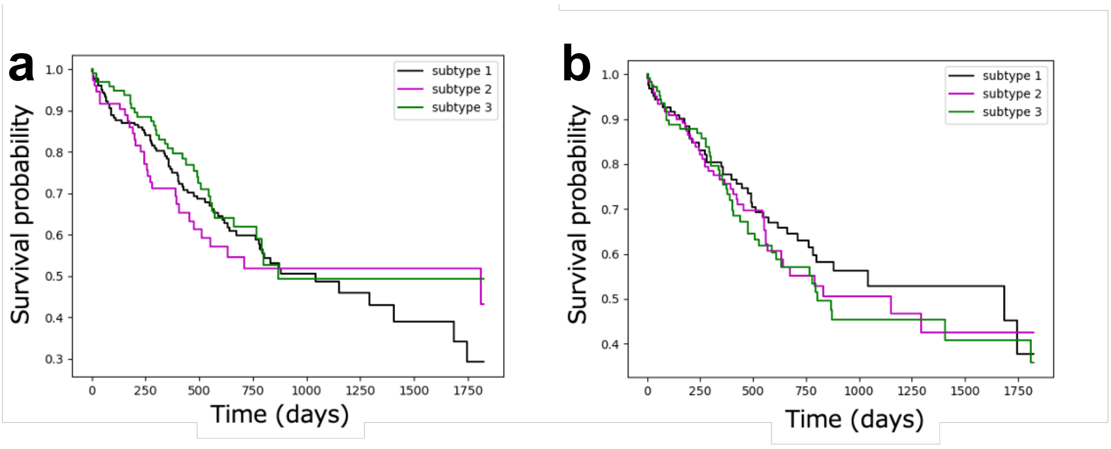

Figure S4. (a) Kaplan-Meier survival probability curves of patients identified by the iNMF method using gene expression data alone. The log-rank test p-value = 0.35 for the identified three subtypes. The log rank test p-value between two subtypes; 0.93 (subtype 1 vs 2) > 0.05, 0.45 (subtype 2 vs 3) > 0.05, and 0.31 (subtype 2 vs 3) > 0.05. (b) Kaplan-Meier survival probability curves of patients identified by the iNMF method using multi-omics data. The log-rank test p-value = 0.32 for the identified three subtypes. The log-rank test p-value between two subtypes; 0.53 (subtype 1 vs 2) > 0.05, 0.34 (subtype 2 vs 3) > 0.05, and 0.78 (subtype 2 vs 3) > 0.05.

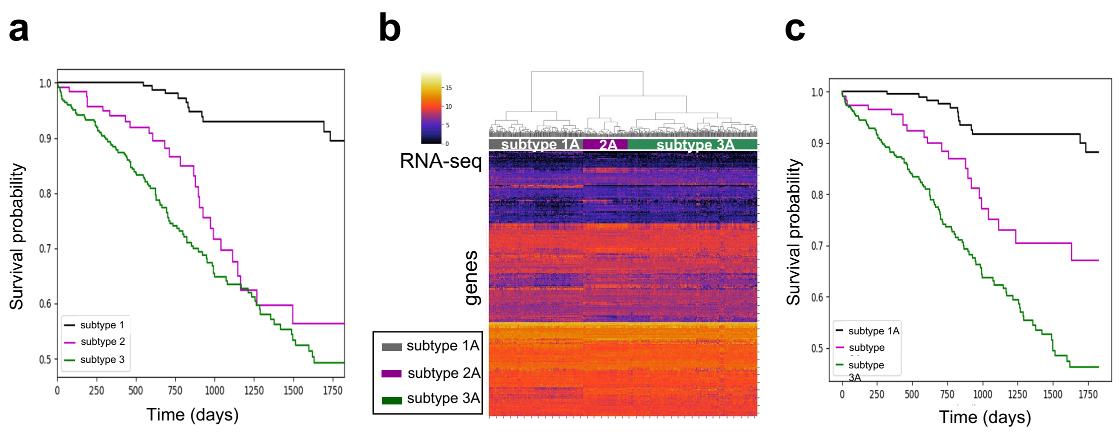

Figure S5. Analysis in the LUNG dataset. (a) Kaplan-Meier survival probability curves of patients for the identified ECv-based subtypes. The log-rank test p-value = 1.3e-14 for the identified three subtypes. The log rank test p-value between two subtypes; 3.1e-08 (subtype 1 vs 2) < 0.05, 9.6e-15 (subtype 1 vs 3) < 0.05, and 0.099 (subtype 2 vs 3) > 0.05. (b) Heatmap of the RNA-seq value matrix. (c) Kaplan-Meier survival probability curves of patients for the identified RNA-seq based subtypes. The log-rank test p-value = 1.5e-15 for the identified three subtypes. The log rank test p-value between two subtypes; 9.8e-05 (subtype 1A vs 2A) < 0.05, 5.5e-15 (subtype 1A vs 3A) < 0.05, and 3.6e-03 (subtype 2A vs 3A) < 0.05.

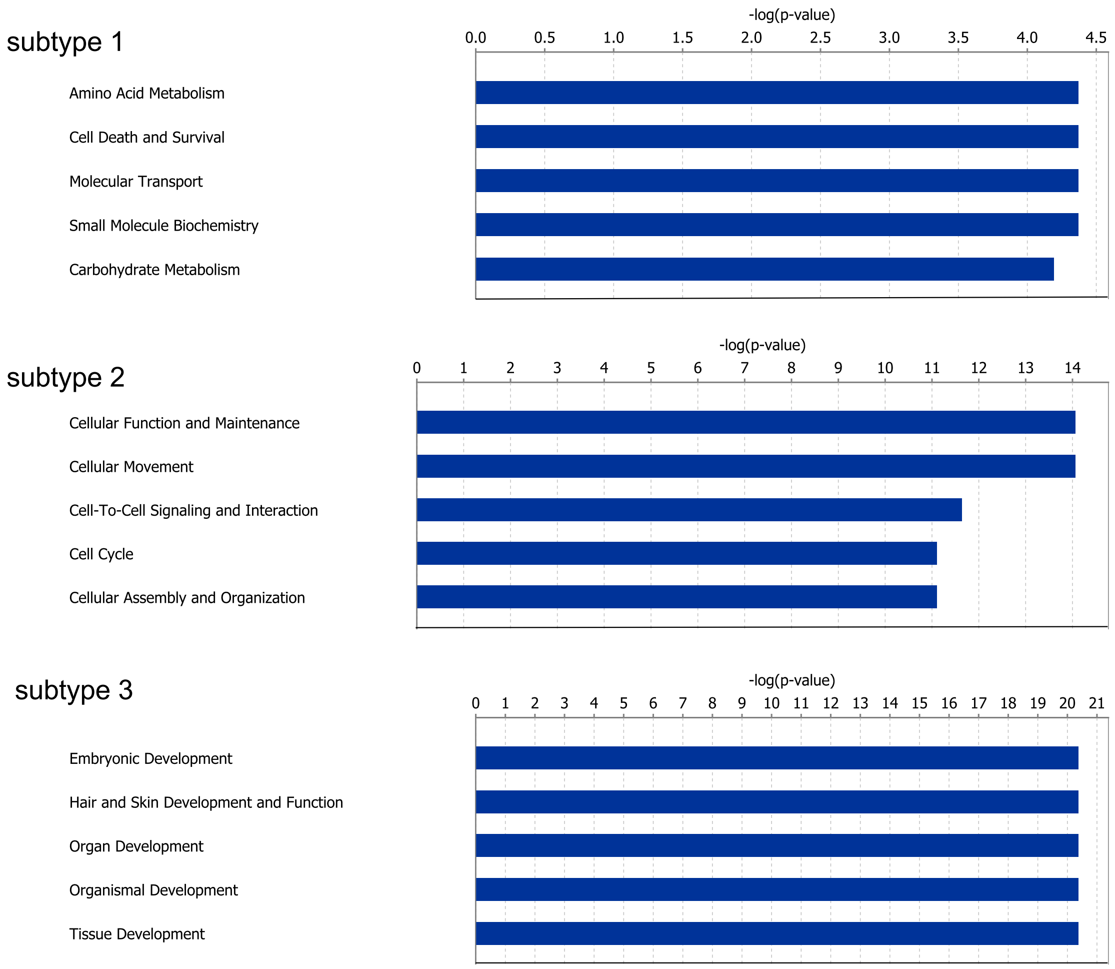

Figure S6. The top five terms of biological functions in LUNG dataset.

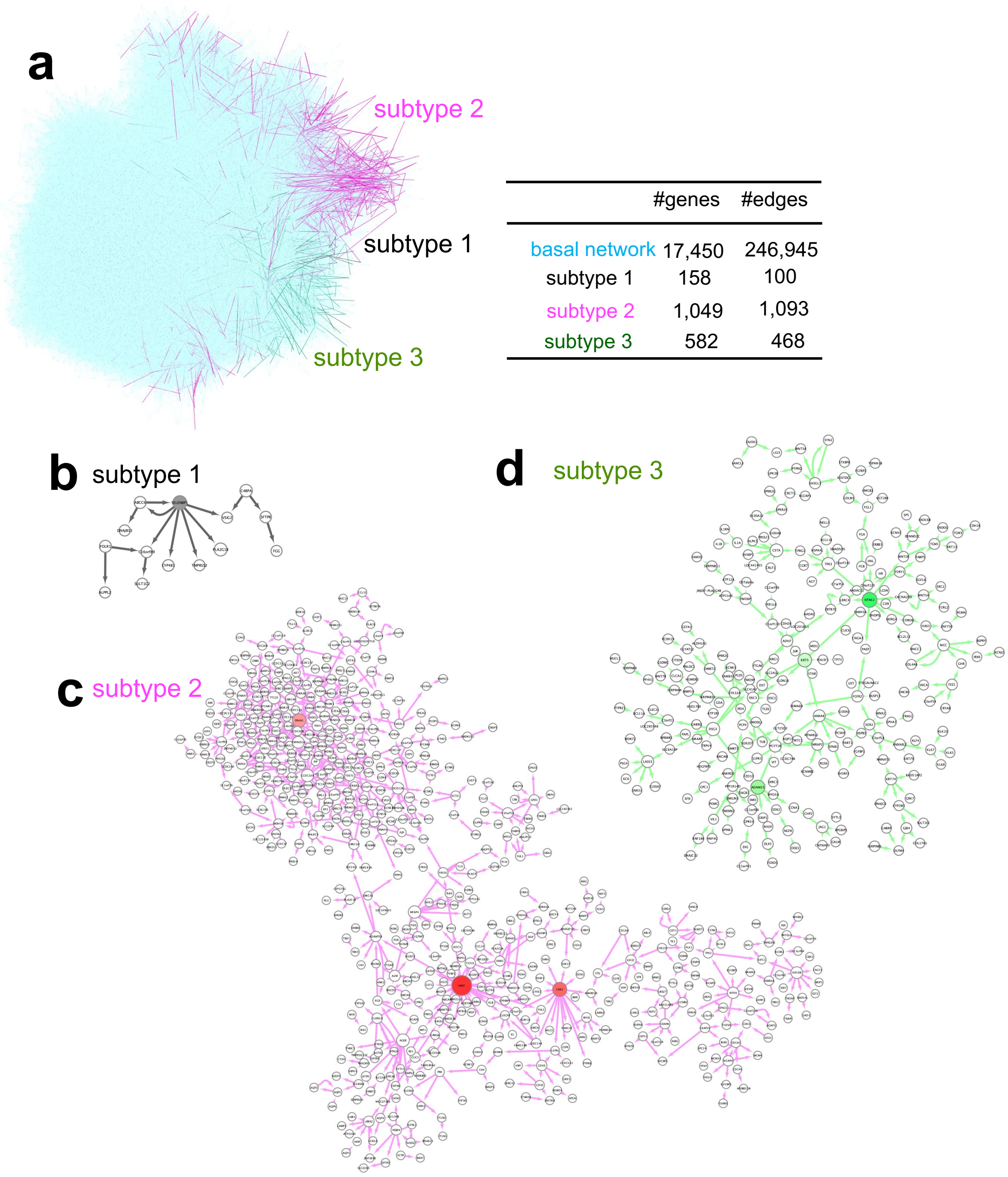

	#genes	#edges
basal network	17,450	246,945
subtype 1	158	100
subtype 2	1,049	1,093
subtype 3	582	468

Figure S7. Visualization of subtype-specific subnetworks in the LUNG datasets.

(a) Subnetworks of subtype-specific edges were highlighted with the basal network (blue). (b-d) The biggest component in the subnetwork of subtype-specific edges in each subtype. Edges and nodes were colored by each subtype; subtype 1 (gray), subtype 2 (magenta), and subtype 3 (green). Colored nodes were hub nodes in each subtype and the color gradient represents the outdegree of hubs.

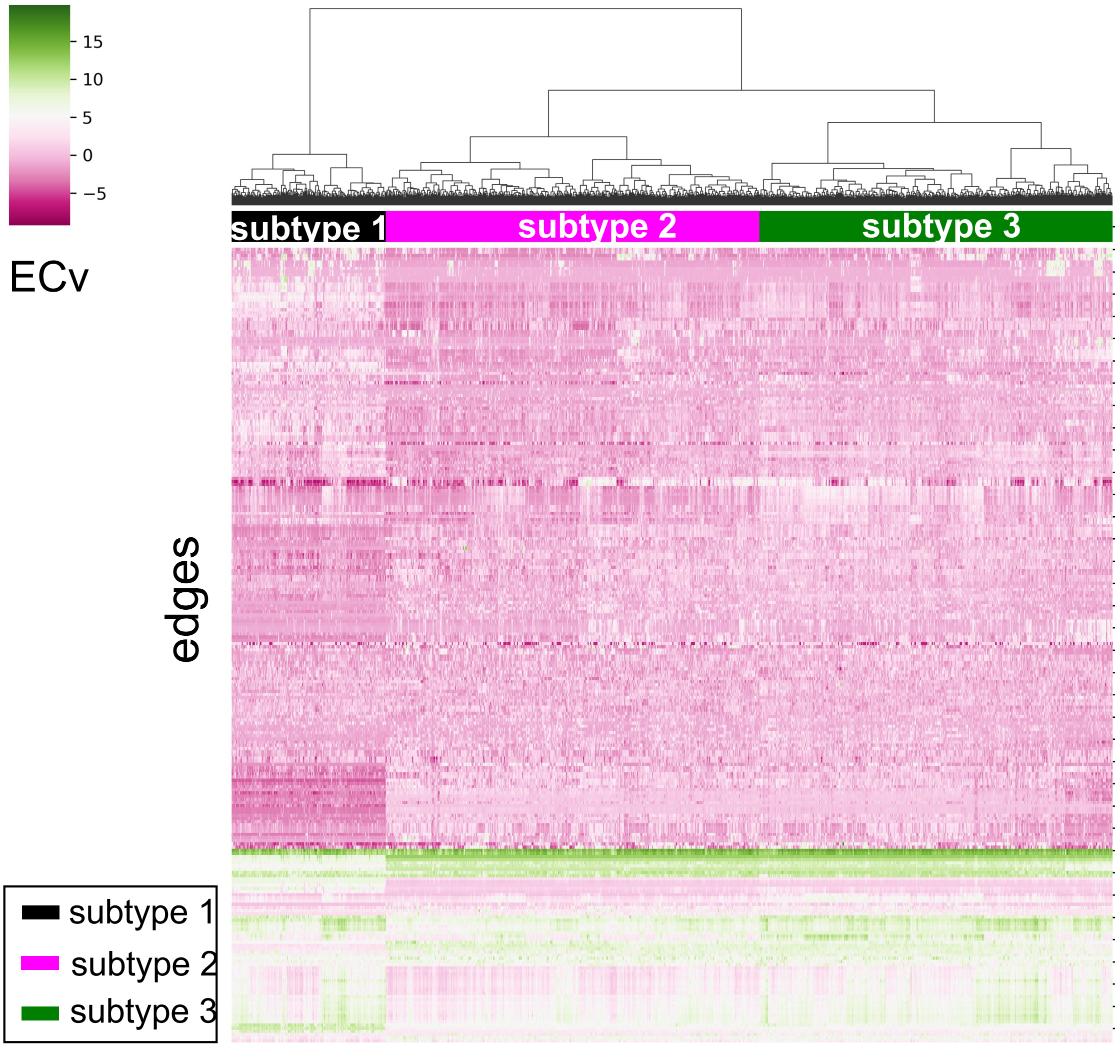

Figure S8. Heatmap showing hierarchical clustering for the ECv matrix in the BRCA dataset.

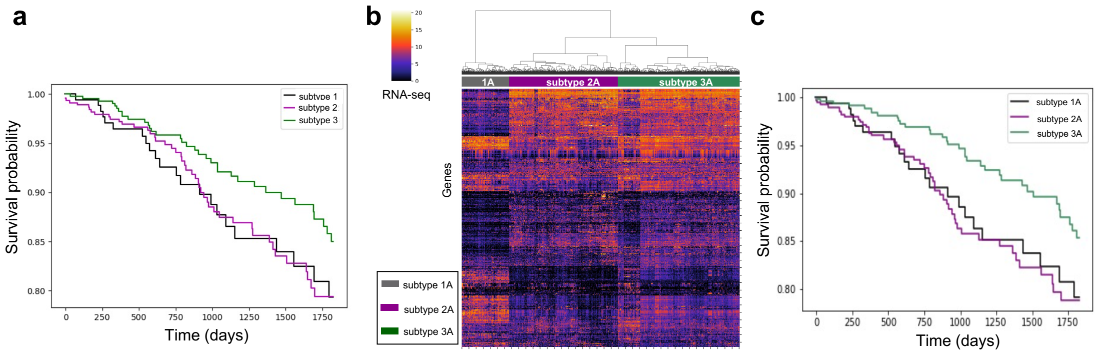

Figure S9. Analysis in the BRCA dataset. (a) Kaplan-Meier survival probability curves of patients for the identified ECv-based subtypes. The log-rank test p-value = 0.0504 for the identified three subtypes. The log rank test p-value between two subtypes; 0.88 (subtype 1 vs 2) > 0.05, 0.11 (subtype 1 vs 3) > 0.05, and 0.08 (subtype 2 vs 3) > 0.05. (b) Heatmap of the RNA-seq value matrix. (c) Kaplan-Meier survival probability curves of patients for the identified RNA-seq based subtypes. The log-rank test p-value = 0.005 for the identified three subtypes. The log rank test p-value between two subtypes; 0.87 (subtype 1A vs 2A) > 0.05, 0.05(subtype 1A vs 3A), and 0.01 (subtype 2A vs 3A) < 0.05.

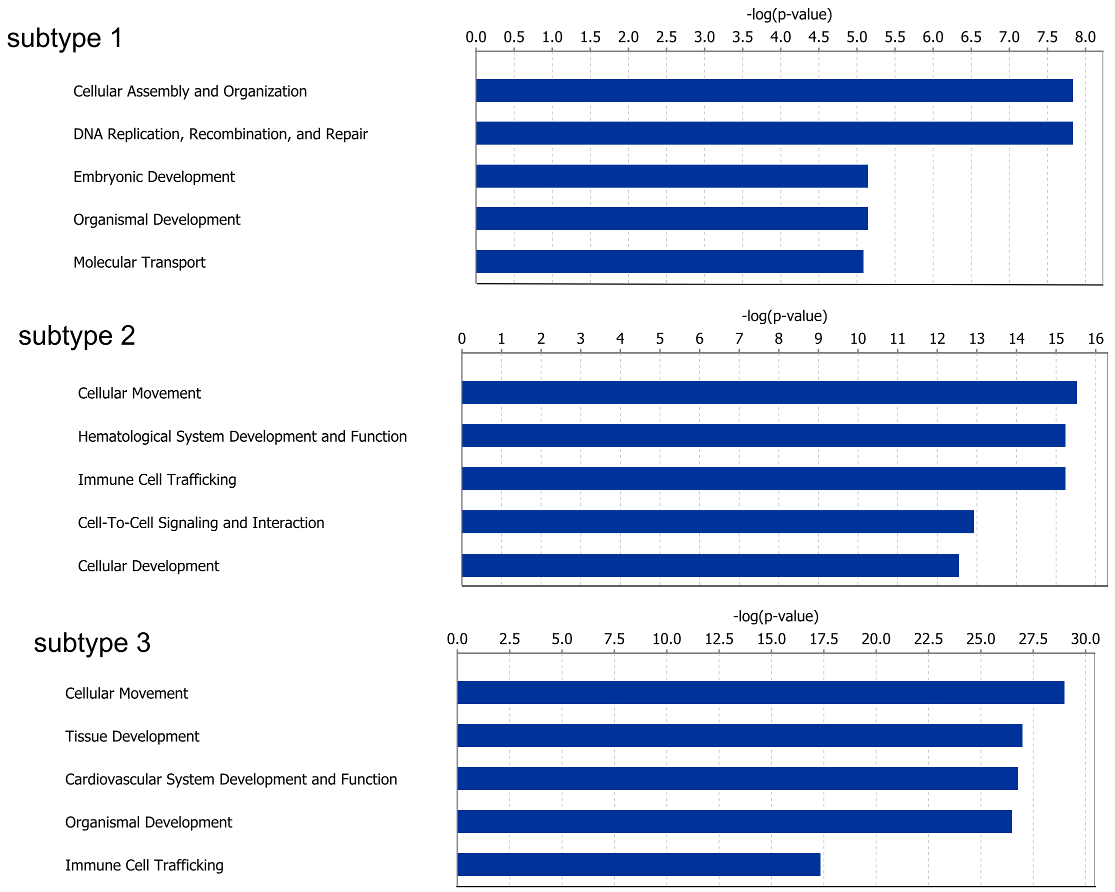

Figure S10. The top five terms of biological functions in BRCA dataset.

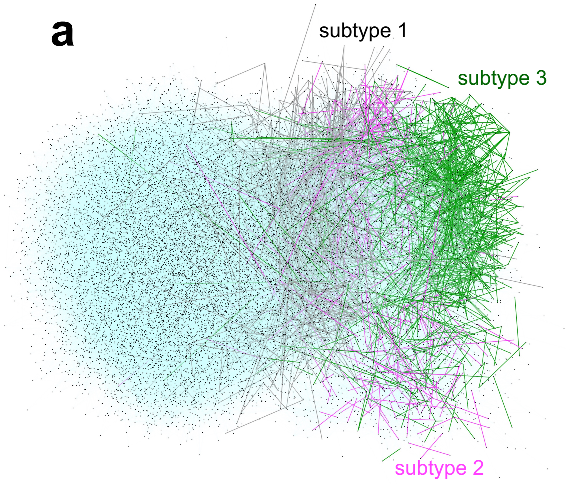

	#genes	#edges
basal network	17,450	236,558
subtype 1	1,052	1,004
subtype 2	463	332
subtype 3	953	979

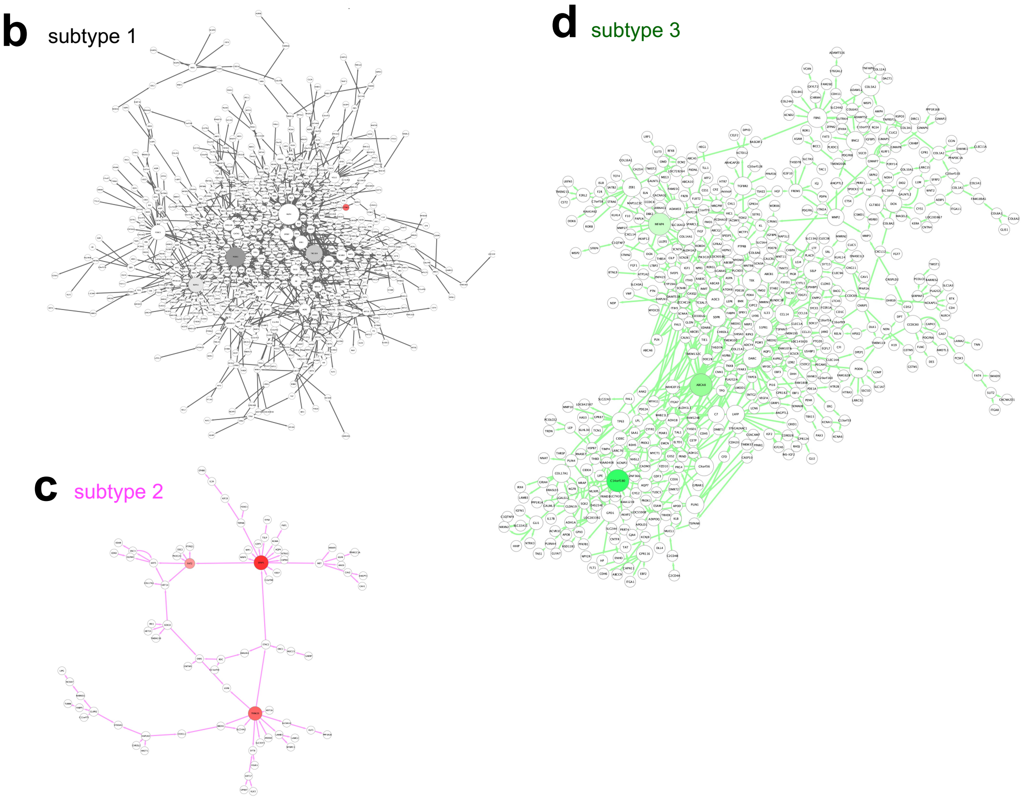

Figure S11. Visualization of subtype-specific subnetworks in BRCA datasets.

(a) Subnetworks of subtype-specific edges were highlighted with the basal network (blue). (b-d) The biggest component in the subnetwork of subtype-specific edges in each subtype. Edges and nodes were colored by each subtype; subtype 1 (gray), subtype 2 (magenta), and subtype 3 (green). Colored nodes were hub nodes in each subtype and the color gradient represents the outdegree of hubs.

Reference

1. Krämer, A. *et al.* (2014) Causal analysis approaches in Ingenuity Pathway Analysis. *Bioinformatics*, **30**, 523–530.
2. Shannon, P. *et al.* (2003) Cytoscape: A software environment for integrated models of biomolecular interaction networks. *Genome Res.*, **13**, 2498–2504.
3. Goldman, M. *et al.* (2019) The UCSC Xena platform for public and private cancer genomics data visualization and interpretation. *bioRxiv*, 326470.
4. Tanaka, Y. *et al.* (2020) System-Based Differential Gene Network Analysis for Characterizing a Sample-Specific Subnetwork. *Biomolecules*, **10**, 306.
5. Tamada, Y. *et al.* (2011) Estimating genome-wide gene networks using nonparametric bayesian network models on massively parallel computers. *IEEE/ACM Trans. Comput. Biol. Bioinform.*, **8**, 683– 697.
